# East Asian heatwaves driven by Arctic-Siberian warming

**DOI:** 10.1038/s41598-022-22628-9

**Published:** 2022-10-27

**Authors:** Jeong-Hun Kim, Seong-Joong Kim, Joo-Hong Kim, Michiya Hayashi, Maeng-Ki Kim

**Affiliations:** 1 Department of Atmospheric Sciences, Kongju National University, Gongju, 32588 Republic of Korea; 2 Division of Atmospheric Sciences, Korea Polar Research Institute, Incheon, 21990 Republic of Korea; 3 Earth System Division, National Institute for Environmental Studies, Tsukuba, Ibaraki 305-8506 Japan

**Keywords:** Climate sciences, Atmospheric science, Climate change

## Abstract

This study investigates the contributing factors of East Asian heatwaves (EAHWs) linked to the Arctic-Siberian Plain (ASP) over the past 42 years (1979–2020). EAHWs are mainly affected by two time scales of variabilities: long-term externally forced and interannual variabilities. The externally forced EAHWs are attributed to the increasing global warming trend, while their interannual variability is related to the circumglobal teleconnection (CGT) and the ASP teleconnection patterns. In addition to the CGT, the Rossby wave energy originating from the ASP propagates to East Asia through the upper troposphere, amplifying the EAHWs. The stationary high pressure in the ASP is generated by vorticity advection in the upper troposphere. Enhanced surface radiative heating and evaporation on the ASP surface increase the specific humidity and temperature, amplifying the thermal high pressure via positive water vapor feedback. Thermal high-pressure amplified by land–atmosphere interactions in the ASP during the peak summer season leads to EAHWs by the propagation of stationary Rossby wave energy. The results indicate that our enhanced understanding of the ASP teleconnection can improve forecasting of the EAHWs not only on a sub-seasonal time scale but also in future projections of global climate models.

## Introduction

In recent decades, heatwaves have strengthened and occurred more frequently in East Asia (EA), including in China, Korea, and Japan^1^. Especially in the summers of 2013, 2016, and 2018, unprecedentedly severe heatwaves have occurred over the Korean Peninsula^2,3^. The maximum temperature reached 41.0 °C in Hongcheon in Korea in 2018, resulting in the longest recorded heat wave, lasting approximately 31.5 days^3^. This event persisted for over than a month, leading to the death of 48 people and heat illness affecting approximately 4000 people^3,4^. The intensity and frequency of heatwaves and their associated socioeconomic damages have been increasing; hence, many studies have attempted to identify their causes. However, the mechanisms of recent intense and frequent heatwaves have become more complicated under anthropogenic warming^1,3,5–7^.

Previous studies have suggested that the summer East Asian heatwaves (hereafter, EAHWs) are affected by various large-scale teleconnections and climatic factors in the low and middle latitudes^8–12^. In general, the EAHW is significantly affected by the Pacific-Japan (PJ) pattern, which is a meridionally oriented circulation variability forced by the western tropical Pacific warm pool^13^. This PJ pattern is mainly induced by strong convection in the South China Sea, transporting heat and water vapor northward^14^. A recent study revealed that temperature advection In the lower troposphere strengthened by the PJ pattern was the main cause of heatwaves in Korea and Japan^15^. The second pattern, suggested by Ding and Wang^16^, is the circumglobal teleconnection (CGT) pattern (or Silk Road pattern), which is an atmospheric wave propagation elongated to the east–west direction in mid-latitudes. Kim et al.^17^ suggested that diabatic heating in northwestern India can intensify heatwaves over the Korean Peninsula by strengthening the CGT pattern.

Recent studies have also reported that the teleconnection patterns in high latitudes (e.g., North Atlantic Oscillation/Arctic Oscillation (NAO/AO), Scandinavian pattern (SCAND), and North Atlantic–Eurasian (AEA) pattern) can affect the EAHW. Deng et al.^18^ and Yoon et al.^19^ reported that the variability of the NAO associated with the sea surface temperature (SST) in the North Atlantic amplified the Rossby wave to propagate across the Eurasian continent, resulting in the recently increased EAHW. Choi et al.^20^ suggested that the amplified SCAND pattern, a decadal change in interannual variability in the mid-1990s, plays a more critical role than the CGT pattern in affecting the EAHW in the current period. Li et al.^21^ suggested that reducing the Barents Sea ice concentration (SIC) can trigger favorable atmospheric circulation associated with hot drought events in summer in northeastern China. Zhang et al.^22^ suggested that the wintertime negative Arctic Oscillation (AO) affects the tripolar SST anomaly over the North Atlantic and can persist until the following summer. These tripolar SST anomalies can amplify the propagation of Rossby wave energy to EA, resulting in heat waves over the Yangtze–Huaihe River Basin in China^23^.

Xu et al.^24^ named the zonally elongated wave propagation in high latitudes (around 60°N) the British–Baikal Corridor (BBC) pattern. The BBC pattern affects the summer surface air temperature (SAT) and precipitation anomalies along its wave pathway (i.e., west of the British Isles, the Baltic Sea, western Siberia, and Lake Baikal). Li et al.^25^ suggested that the combined effect of CGT and BBC patterns can favorably modulate middle latitude atmospheric circulation to more severe EAHWs. Using self-organizing map (SOM) clustering on a daily time scale, Lee et al.^26^ and Kim et al.^12^ investigated the interannual variability of heatwaves in Korea and found several high-latitude teleconnection centers in each SOM cluster associated with heatwaves.

In these studies of high-latitude teleconnection for the EAHW, the Arctic-Siberian Plain (ASP) was often the center of action for the teleconnection. Although the warming of the ASP was prominent in the BBC pattern suggested by Xu et al.^24^, the mechanism of teleconnection from the ASP to EAHW on the daily time scale has not been investigated. A SOM clusters described in Lee et al.^26^ and Kim et al.^12^, the ASP was clearly shown to be one of the centers of action for the teleconnection; however, the relationship between the warming of the ASP and the extreme EAHW was unexplored. Although many attempts to investigate the high-latitude teleconnections that induce the EAHW have been made, its mechanism on the daily time scale has not yet been clarified. Therefore, we hypothesize that remote forcing over the ASP may significantly trigger extreme EAHWs. This study conducts an in-depth analysis of the EAHW associated with the high-latitude ASP teleconnection, after removing the CGT pattern from the reanalysis data (see details in method section).

## Results

### Characteristics of East Asian heatwaves and related atmospheric circulation patterns

**Figure 1:**
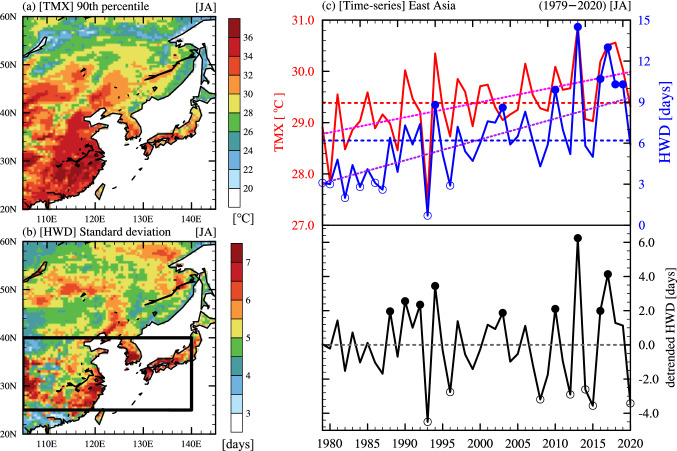
Climatological features of maximum temperature (TMX) and heatwave days (HWD) in East Asia. (**a**) 90th percentile of daily TMX (unit: °C) (1979–2020) in peak summer (July–August), and (**b**) standard deviation of HWD (unit: days). The black rectangle indicates the area of interest in East Asia where the standard deviations of HWD are high. (**c**) Time series of TMX (red solid line), HWD (blue solid line), and detrended HWD (black solid line) in the black rectangle area in (**b**). The open and closed circles indicate heatwave years in East Asia (HWY-EA) and non-heatwave years in East Asia (NHWY-EA), identified from the HWD with the trend (blue) and detrended HWD (black), respectively. The horizontal dashed lines indicate the climatological mean and the pink and purple dotted lines indicate the trends.

Figure 1 shows the climatic characteristics of maximum temperature (TMX) and heatwave days (HWD; see the Methods section for details about the definition of the HWD) in EA. The 90th percentile of TMX is high in South and East China, the Korean Peninsula, and western Japan, with an average TMX of 33.2 °C (Fig. 1a). HWD also frequently occurred where the 90th percentile of TMX is high, with high standard deviations (Fig. 1b). The area-weighted average of TMX and HWD shows notable interannual variability with a significant increasing trend in EA (Fig. 1c). During the analyzed period, the averages of TMX and HWD were 29.4 °C and 6.2 days, respectively. TMX increased from 28.8 to 29.9 °C, and HWD increased from 3.1 days to 9.3 days, indicating a three-fold increase throughout the period. HWD shows a steep increasing trend during the period, as in previous results^20,27,28^. To examine the interannual variability affecting the EAHW, we removed the linear trend of the HWD (Fig. 1c bottom). The detrended HWD reveals the periodic changes in the variability with a more significant fluctuation in the recent decade, in which the standard deviation changed from 1.89 (before the 2000s) to 2.56 (after the 2000s). An increasing standard deviation after the 2000s implies that more extreme heat wave events have occurred in East Asia. In this study, we classified the heatwave years (HWY-EA) and non-heatwave years (NHWY-EA) based on the threshold of ± 0.8 sigma of the raw HWD and the detrended HWD in EA (Table 1). Most of the HWY-EA (with a trend) occurred after the 2000s, except for 1994. However, the HWY-EA (detrended) associated with the interannual variability occurred evenly over the entire analysis period. Interestingly, heatwaves that occurred in 1994, 2003, 2010, 2013, 2016, and 2017 (a total of 6 years) were caused by the overlapping of the externally forced and interannual variabilities.

**Table 1:** Heatwave and non-heatwave years in East Asia (HWY-EA and NHWY-EA) were defined by the ± 0.8 standard deviation thresholds of the HWD with the trend and detrended HWD, respectively.

Thresholds: ± 0.8 sigma of HWD		Number of years
HWY-EA (with trend)	1994, 2003, 2010, 2013, 2016, 2017, 2018, 2019	8
NHWY-EA (with trend)	1979, 1980, 1982, 1984, 1986, 1987, 1993, 1996	8
HWY-EA (detrended)	1988, 1990, 1992, 1994, 2003, 2010, 2013, 2016, 2017	9
NHWY-EA (detrended)	1993, 1996, 2008, 2012, 2014, 2015, 2020	7

### Relationship between the variability in the Arctic-Siberian plain and East Asian heatwaves

**Figure 2:**
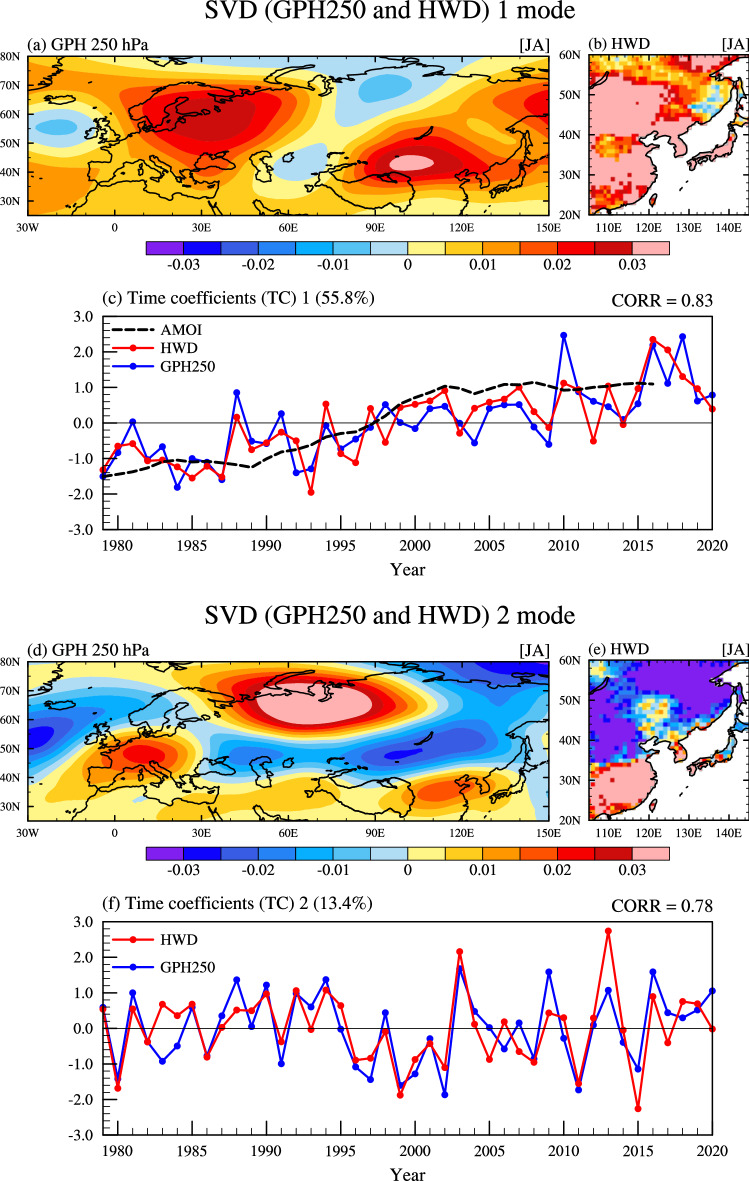
SVD mode 1 and mode 2 of geopotential height at 250 hPa (GPH250) and heatwave days (HWD). (**a**)–(**c**) First and (**d**)–(**f**) second coupled modes of SVD. From July to August, the coupled patterns of (**a** and **d**) GPH250 and (**b** and **e**) HWD in East Asia. (**c** and **f**) The standardized time series of the SVD time coefficients (TC) for GPH250 (blue line) and HWD (red line). The black dashed line in (**c**) indicates the AMO index.

To confirm the covariability between atmospheric circulation patterns in the Northern Hemisphere in summer and HWD in EA, we performed a singular value decomposition (SVD) analysis (Fig. 2). The first mode explains 55.8% of the total covariance, and the correlation coefficient between the two time coefficients (hereafter, TC1) is 0.83, which is significant at a 99% confidence level. The geopotential height (GPH) at 250 hPa (hereafter, GPH250) shows a wave train pattern, originating from the North Atlantic Ocean and propagating to Northern China and EA through the middle latitude continent (Fig. 2a and supplementary Fig. S1). This atmospheric circulation pattern resembles a linear trend pattern of the upper troposphere (not shown) and the Northern Hemisphere atmospheric circulation pattern associated with global warming demonstrated in previous studies^20,29,30^. Moreover, the HWD pattern of the EA region coupled with the atmospheric circulation pattern in the upper troposphere shows strong positive anomalies for the entire EA region (Fig. 2b). TC1, which shows a distinct linear increasing trend, also resembles the time series of the Atlantic Multidecadal Oscillation (AMO) index with the same phase (Fig. 2c). This result is consistent with the variability of low-frequency HWD in EA presented by Choi et al.^20^, indicating that the first mode of SVD, representing long-term variabilities such as global warming and AMO, affects the variability of HWD in the entire EA region. These results closely resemble previous studies on the long-term variability of the EAHW^23,31–34^. However, Mann et al.^35^ suggested that the AMO is an artifact of pulses of volcanic activity during the preindustrial period, and there is no clear evidence for internal multidecadal oscillations in the climate system. However, this study does not analyze the multidecadal oscillations, such as AMO, in detail due to the limitation of the analysis period.

In the second mode of SVD, which explains 13.4% of the total covariance, the GPH250 pattern shows a distinct center of action in the ASP region (Fig. 2d). In addition, the second mode of the HWD pattern, coupled with the GPH250 pattern, shows a robust north–south dipole that switches along ~ 35°N (Fig. 2e). The correlation coefficient between the two time coefficients of the second mode (hereafter, TC2-GPH250 and TC2-HWD) is 0.78, which is significant at a 99% confidence level (Fig. 2f). The interannual variability in TC2-HWD has slightly increased since the 2000s from 0.85 to 1.15, as indicated in detrended HWD of Fig. 1c. In addition, the HWY and NHWY related to the ASP, defined based on the threshold of ± 0.8 sigma of TC2-HWD (hereafter, HW-ASP and NHW-ASP; Table 2), are consistent with the HWY-EA (detrended) caused by the interannual variability in EA in Table 1, indicating that the atmospheric circulation pattern originating from the ASP contributes significantly to EAHW, related to interannual variability. Furthermore, EAHW originating from the ASP are quite in phase with the HWY-EA (with a trend), showing that recent EAHW may have occurred more intensely and frequently due to the combined effect of the increasing trend with interannual variability.

**Table 2:** Heatwave and non-heatwave years originating from the ASP (HW-ASP and NHW-ASP) were defined by the ± 0.8 standard deviation thresholds of the 2nd SVD mode time coefficients (TC2) for GPH250 and HWD.

Thresholds: ± 0.8 sigma of TC2		Number of years
HW-ASP (GPH250)	1981, 1988, **1990**, **1992**, **1994**, **2003**, 2009, **2013**, **2016**, 2020	10
NHW-ASP (GPH250)	**1980**, 1983, 1991, **1996**, **1997**, **1999**, 2000, **2002**, **2008**, **2011**, **2015**	11
HW-ASP (HWD)	**1990**, **1992**, **1994**, **2003**, **2013**, **2016**	6
NHW-ASP (HWD)	**1980**, 1986, **1996**, **1997**, **1999**, **2002**, 2005, **2008**, **2011**, **2015**	11

Underlined years indicate those in phase with the HWY from detrended HWD.

Bolded years mean those in step with the HW-ASP (GPH250) and HW-ASP (HWD).

**Figure 3:**
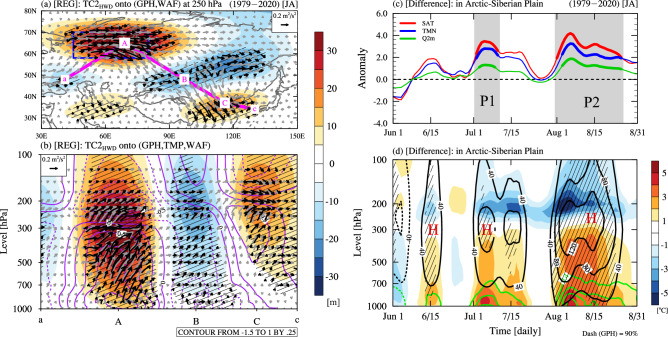
Regression and composite pattern associated with the Arctic-Siberian Plain (ASP). (**a** and **b**) Horizontal and vertical regression patterns between the 2nd SVD mode time coefficients (TC2) for heatwave days (HWD) (TC2-HWD) and geopotential height (GPH; shading; units: m), wave activity flux (WAF; vector; unit: m^2^/s^2^), and air temperature (TMP; purple line; unit: °C). The blue rectangle in (**a**) indicates the ASP region (58°N–70°N, 45°E–80°E). The solid pink line indicates a Rossby wave pathway. The black vectors indicate statistically significant values at a 90% confidence level. (**c** and **d**) Composite difference between heatwaves associated with ASP (HW-ASP) and non-heatwaves associated with ASP (NHW-ASP) for (**c**) 7-day running mean surface air temperature (SAT; red line; unit: °C), minimum temperature (TMN; blue line; unit: °C), and 2 m specific humidity (Q2m; green line; unit: g/kg) and (d) TMP (shading; unit: °C), GPH (black contour; units: m), and specific humidity (green contour; unit: g/kg). The thick solid line in (**c**) and the hatched patterns in (**d**) indicate the values statistically significant at a 90% confidence level. Gray shading indicates period 1 (P1; July 02–July 10) and period 2 (P2; August 01–Augusy 25).

The dynamic mechanism of the HW-ASP affecting the EAHW was examined using the regression pattern between TC2-HWD and atmospheric variables (Fig. 3a and b). In the upper troposphere, the Rossby wave clearly propagates from the ASP to EA across the Eurasian continent (pathway through A to C in Fig. 3a). The vertical cross-section along the pathway of Rossby wave energy propagation shows that the positive temperature anomalies strongly developed from the lower to the middle troposphere in the ASP, causing anomalous thermal high pressure. In this study, the thermal high-pressure anomalies amplified by surface heating formed a dome, which was defined as a heat dome. At the same time, the wave activity flux (WAF) propagated strongly upward from the lower troposphere, amplifying the spatial height amplitude and the upper troposphere Rossby wave energy propagation towards the EA region.

To investigate the physical mechanism of the heat dome, which vertically develops in the ASP, we examined the composite differences in temporal changes in various surface and atmospheric variables between HW-ASP and NHW-ASP (Fig. 3c and d). The variables were analyzed after applying a 7-day running mean to eliminate high-frequency variability. The SAT is related to the daily minimum air temperature (TMN) and specific humidity. When the HW-ASP occurred, the SAT in the ASP significantly increased at the beginning of July. Concurrently, it shows that TMN and specific humidity also increased. Diurnal variations in surface variables show that the increase in evaporation due to an increase in SAT causes an increase in specific humidity during the daytime (supplementary Fig. S2). The warm and moist air caused by an increase in water vapor, which induces the greenhouse effect, can decrease radiative cooling during the nighttime, thereby increasing TMN. The lower troposphere, which does not have sufficient radiative cooling during the nighttime, is further heated by intense insolation the following day, resulting in a more significant increase in SAT. According to the Clausius–Clapeyron equation, a warmer atmosphere can contain more water vapor. Therefore, the variations in SAT and specific humidity in the ASP are controlled by the positive water vapor feedback, which causes the heat dome to amplify the thermal high-pressure and elevated GPH surfaces.

To identify what caused the initial SAT increase, we further examined the surface energy budgets at the time when the surface temperature rose markedly in the ASP (Table 3). We divided the period into two interval based on the SAT evolution in the gray shaded areas in Fig. 3c, wherein the SAT markedly increased in July and August. Incoming (outgoing) net shortwave (longwave) radiation to (from) the surface occurred due to the anomalous high pressure in the upper troposphere during the two periods. The latent heat release from the surface to the atmosphere was significant with the magnitude of approximately 10.36 and 5.45 W m^−2^ in the P1 and P2 periods, respectively. There was no significant increase in sensible heat flux due to surface wetness characteristics because the ASP region is covered by extensive ill-drained swamps and floodplains. Therefore, the ASP area allows for substantial evaporation, and the latent heat flux can be higher than the sensible heat flux. Thus, the greenhouse effect due to abundant water vapor in the atmosphere increases the downward longwave radiation to approximately 7.45 and 10.23 W m^−2^ in the P1 and P2 periods, which contributes to the warming of the ASP.

**Table 3:** Composite difference between HW-ASP (HWD) and NHW-ASP (HWD) for the surface energy budget components (units: W m^−2^, positive: downwards, negative: upwards) over the ASP in P1 (July 2–July 10) and P2 (August 1–August 25) periods.

Period	Net shortwave radiation	Net longwave radiation	Net sensible heat flux	Net latent heat flux	Downward longwave radiation
P1 (7/2 ~ 7/10)	28.77**	− 9.03**	− 1.04	− 10.36**	7.45*
P2 (8/1 ~ 8/25)	17.85***	− 6.19**	− 2.02	− 5.45**	10.23***

***: 99%, **: 95%, *: 90% confidence levels.

**Figure 4:**
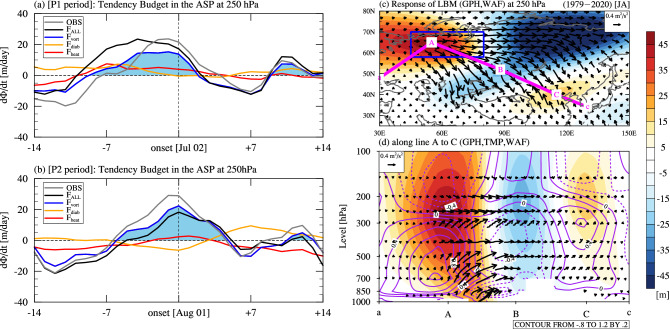
Quasi-geostrophic geopotential tendency (QG tendency) budget analysis and model experiments. Time series of the 7-day running mean QG tendency budget (units: m/day) in the ASP at 250 hPa for (**a**) P1 and (**b**) P2 periods. The solid gray line indicates the geopotential height (GPH) tendency obtained from reanalysis data. Black, red, orange, and solid blue lines indicate the QG tendency budget induced by the total forcing (F_ALL_), temperature advection (F_heat_), diabatic heating (F_diab_), and vorticity forcing (F_vort_), respectively. The shading in (**a**) and (**b**) indicates a positive vorticity forcing period. (**c** and **d**) Same as in Fig. 3a and b but for the atmospheric responses to the vorticity tendency and diabatic heating (Q1) forcings in the linear baroclinic model (LBM) experiment. These steady forcings in the LBM are derived from the daily relative vorticity tendency and Q1 over the Arctic-Siberian Plain (ASP) region from the surface level to 10 hPa with a temporal average from days − 3 to 0 (onset) during the P1 and P2 periods in (**a**) and (**b**).

To identify the causes of abnormal high pressure in the ASP, we performed a budget analysis of each forcing that affects the development of anomalous high pressure using the quasi-geostrophic geopotential tendency (hereafter, QG tendency) equation budget analysis (Fig. 4a and b). The solution of the QG tendency budget shows the sum of all forcings (i.e., vorticity advection, temperature advection, and diabatic heating). The solution is similar to the GPH tendency calculated using reanalysis data, indicating that the obtained QG tendency budget is valid for analyzing the contribution of each forcing. The forcing of vorticity advection explains most of the total GPH change in the upper troposphere when anomalous high pressure occurs in the ASP. However, the forcings of temperature advection and diabatic heating play relatively minor roles in the geopotential tendency in the upper troposphere.

### Model experiments

Our analyses thus far indicated that the anomalous high pressure that develops in the ASP because of vorticity advection in the upper troposphere is amplified by the increase in local SAT due to the increase in latent heat fluxes driven by specific humidity in the lower troposphere. We conducted a linear baroclinic model (LBM)^36^ experiment (see details in the Method section) to confirm whether the steady forcings for the relative vorticity and temperature in the ASP region generate and amplify the atmospheric circulation pattern related to HW-ASP (Fig. 4c and d). This experiment prescribed vorticity tendency and diabatic heating in the ASP region with a temporal averaging of − 3 to 0 (onset) days of the P1 and P2 periods based on the composite differences between HW-ASP and NHW-ASP (Supplementary Fig. S3). The maximum negative value of vorticity forcing, which is dominated by advection, appears in the upper troposphere (supplementary Figs. S3a and c). Diabatic heating (hereafter, Q1) is shown in the near-surface due to surface heating; however, the lower to upper troposphere shows diabatic cooling due to abnormally high pressure (supplementary Figs. S3b and d). We removed the zonal component in the calculation for the WAF in the model to stress only a meridional response, as the results of the LBM experiment predominantly represent a robust zonal propagation of the Rossby wave.

The results of the experiment show that the anomalous high-pressure occurs clearly in the upper troposphere in the ASP and that the Rossby wave propagates to EA via the Eurasian continent, indicating an atmospheric circulation pattern that is favorable to the EAHW. This result is consistent with the HW-ASP pattern, as shown in Fig. 3a. In addition, the numerical experiment shows that the heat dome is strongly developed from the lower troposphere to the middle troposphere in the ASP, and the Rossby wave originating from the lower troposphere propagates to EA along the upper troposphere path, as shown in Fig. 3b. The model results of single forcing experiments indicate that the vorticity forcing in the ASP generates the atmospheric circulation pattern of HW-ASP (supplementary Figs. S4a and b). Simultaneously, diabatic heating intensifies the upward propagation of the Rossby wave, but its contribution is secondary in the LBM experiment (supplementary Fig. S4c and d). Further studies are needed to quantify the role of diabatic heating in the lower troposphere on the EAHW using general circulation models.

## Summary and discussion

In this study, we investigated the atmospheric circulation patterns related to the peak summer (July–August) EAHW for the past 42 years (1979–2020) and the associated teleconnection pattern from the ASP. The EAHW occurs under complex atmospheric circulation patterns driven by both externally forced and interannual variabilities. First, the atmospheric circulation pattern caused by increasing trends is associated with global warming. Second, the atmospheric circulation pattern that affects EAHW shows interannual variability related to the ASP teleconnection pattern.

**Figure 5:**
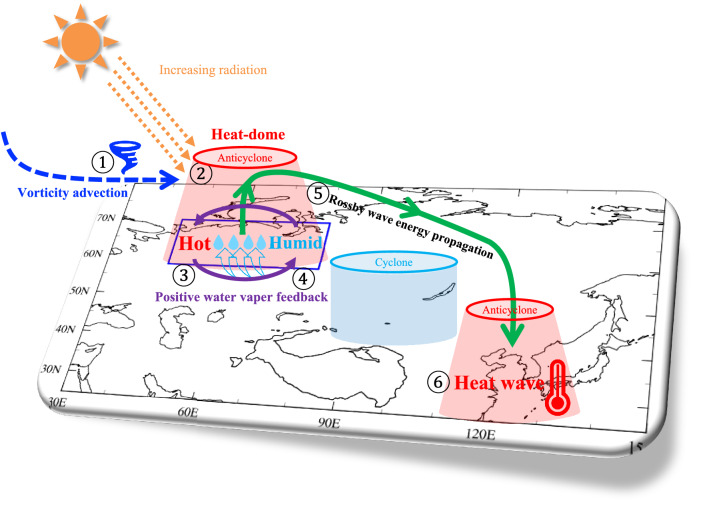
Schematic diagram of East Asian heat waves associated with Arctic-Siberian Plain warming. The blue dashed line indicates vorticity advection, and the solid green and purple lines indicate a Rossby wave energy pathway and the positive water vapor feedback, respectively. The numbers indicate the ordering of the process.

The physical process of heat waves in East Asia associated with ASP warming is summarized in a schematic diagram (Fig. 5). The HW-ASP pattern shows that the Rossby wave amplified from the ASP propagates to East Asia through the upper troposphere, thereby affecting the EAHW. A strong anticyclonic anomaly in the ASP is generated by vorticity advection in the upper troposphere. In addition, an increase in evaporation and radiation heating caused by the intense anticyclonic anomaly in the ASP amplifies a heat dome because of the positive water vapor feedback. Consequently, the strengthened heat dome in the ASP develops thermal high pressure and intensifies the upward propagation of the Rossby wave energy.

Several recent studies have suggested that the AO and variations in the Arctic SIC, contribute to the middle latitude atmospheric circulation field and correlate with the EAHW in summer^14,21,22,37,38^. These studies only suggested a high correlation between the AO, Arctic SIC, and EAHW; however, they did not present the physical mechanisms behind these relationships. The results of this study suggest that the anomalous high pressure, strengthened in the ASP, is expected to play a significant role in decreasing SIC as anticyclonic anomalies in the ASP can enhance the transport of ocean waters and warm air into the BKS. This assumption may imply a high correlation between EAHW and Arctic SIC, but causality remains uncertain. Surface temperatures are near 0 ℃ in summer everywhere over the Arctic Ocean, and surrounding land is generally warmer. Therefore, a correlation between the AO and EAHW would not be expected in July and August because Arctic amplification is not located over the Arctic Ocean^39^. In July and August in the Arctic, the turbulent heat fluxes from the ocean to the atmosphere are almost negligible because the air temperature is higher than the SST. Therefore, in summer, the role of turbulent heat fluxes due to decreased SIC and AO in forming atmospheric conditions should be stated with caution.

The results of this study emphasize that the anomalous high-pressure by vorticity advection in the ASP is amplified by local heating, which amplifies the Rossby wave propagation to EA, leading to favorable atmospheric conditions (i.e., anticyclonic circulation) for heatwaves. The changes in blocking around the Ural Mountain and ASP region may be associated with amplified Arctic warming. However, a deeper analysis is required to prove these assumptions, which is beyond the scope of this study. Recent studies have reported that the acceleration of global warming affects atmospheric circulation, thereby causing a more extreme EAHW^3,40,41^. This study suggests that the Rossby wave energy amplified in the ASP has affected the EAHW with stronger variability in recent years, and these effects are associated with global warming. Our results will help improve forecasting EAHWs not only in a sub-seasonal time scale, but also in future projections of global climate models.

## Methods

### Data

Data for investigating the EAHW during the summer are from the European Center for Medium-Range Weather Forecasts (ECMWF) reanalysis data version 5 (ERA5)^42^ for the period 1979–2020. To analyze the characteristics of the EAHW, we used SAT data for July–August, when heatwaves occurred most frequently. The monthly and daily averaged data were calculated from 1-hourly data, and the horizontal and vertical resolutions were 1° × 1° and 37 pressure levels, respectively.

The AMO is a natural variability occurring in the North Atlantic Ocean with an estimated period of 60–80 years. The AMO index, obtained from the Earth System Research Laboratory in the National Oceanic and Atmospheric Administration (NOAA)^43^, is calculated using an area-weighted average over the North Atlantic in the latitude range 0–70°N and the Kaplan SST dataset (5° × 5° resolution) that is detrended and smoothed over 121 months (https://www.esrl.noaa.gov/psd/data/gridded/data.kaplan_sst.html).

### Definition of HWD

The daily TMX was calculated using 1-hourly SAT data in the EA domain. Defining heatwaves using absolute temperature criteria is impossible because of a significant difference in regional climatological conditions and topography. In this study, we define HWD in EA on each grid cell of TMX based on the 90th percentile method following previous studies^12,23,27^.

### Empirical orthogonal function (EOF)

The interannual variability in atmospheric circulation patterns related to the EAHW is a mixture of distinct atmospheric teleconnection routes toward EA^20,23,25^. Many previous studies have suggested that the CGT pattern affects extreme weather events such as monsoons, heatwaves, and droughts in EA^17,45,46^. To confirm the pattern of heatwaves that originated only in the ASP, we removed the CGT pattern that appears as the second mode of the empirical orthogonal function (EOF) (Supplementary Fig. S5). The EOF analysis of GPH250 over the Northern Hemisphere (30°W–150°E, 20°N–80°N) shows that the second mode explains approximately 13.7% of the total variability, which is consistent with the action center of the CGT pattern. The correlation coefficient between the CGT index and the 2nd mode of GPH250 is 0.32, which is statistically significant at a 95% confidence level. Therefore, except for the second mode, which is classified as the CGT pattern, the other modes were reconstructed using the NCAR Command Language (NCL) built-in function (http://ncl.ucar.edu/Document/Functions/Built-in/eof2data.shtml) and used for analysis.

### Singular value decomposition (SVD)

SVD analysis is a method that is used to find the coupled mode for spatiotemporal variability between two different variables^17,27^. This study performed an SVD analysis to confirm the relationship between HWD in EA in summer and the Northern Hemisphere atmospheric circulation pattern.

### Wave activity flux (WAF)

The WAF is analyzed to trace the propagation path of the Rossby wave energy of the atmospheric circulation pattern affecting the EAHW. Because the WAF analysis is beneficial for determining the propagation path of Rossby wave energy, the three-dimensional WAF was calculated according to the method of Takaya & Nakamura^47^.

### Quasi-geostrophic geopotential tendency budget

The QG tendency equation [Eq. (1)] is calculated following Holton^48^.

1 $$\left\{ {\frac{1}{{f_{0} }}\nabla^{2} + f_{0} \frac{\partial }{\partial p}\left( {\frac{1}{{s_{0} }}\frac{p}{R}\left( {\frac{{p_{0} }}{p}} \right)^{{\frac{R}{{c_{p} }}}} \frac{\partial }{\partial p}} \right)} \right\}\frac{\partial \phi }{{\partial t}} = - {\varvec{V}} \cdot \nabla \left( {\zeta + f} \right) - f_{0} \frac{\partial }{\partial p}\left( { - {\varvec{V}} \cdot \nabla \frac{{\theta_{d} }}{{s_{0} }}} \right) - f_{0} \frac{\partial }{\partial p}\left( {\frac{J}{{s_{0} c_{p} }}} \right)$$

where $f_{0}$ is the Coriolis parameter at 45 $^\circ {\text{N}}$ latitude, ${\varvec{V}}$ is the horizontal wind vector, and $\zeta$ and $f$ are the relative and planetary vorticities, respectively. $R$ is the gas constant of dry air (287 ${\text{Jkg}}^{ - 1} {\text{K}}^{ - 1}$), and $c_{p}$ is the specific heat of constant pressure in dry air ($1004 \;{\text{Jkg}}^{ - 1} {\text{K}}^{ - 1}$). $p_{0}$ is the atmospheric pressure at 1000 hPa, $\theta_{d}$ is the deviation of the temperature in each grid from the mean temperature in the Northern Hemisphere, $s_{0}$ is the average static stability in the Northern Hemisphere, and $J$ is the diabatic heating. Additionally, the QG equation calculated in this study assumed a dry atmosphere without any friction.

The QG tendency is governed by three forcing terms on the right-hand side of Eq. (1) [i.e., vorticity advection (F_vort_), temperature advection (F_heat_), and diabatic heating (F_diab_)]^48^. The budget analysis of the QG tendency of each forcing using the QG tendency equation provides an understanding of the contribution of each forcing to the GPH of a specific region^49,50^. Here, Q1 is calculated based on the thermodynamic energy equation and using the residual term following Yanai et al.^51^.

### Linear baroclinic model (LBM) experiments

To confirm a sustained response of atmospheric patterns of steady forcing affecting the GPH anomaly, we used a dry version of the LBM, which consists of the primitive equation system exactly linearized about a background basic state and has a T42 horizontal resolution with 20 vertical sigma levels^36^. The background field was derived from the climatological mean of ERA5 for 1979–2020 in this experiment. We prescribed the vorticity tendency and diabatic heating with their horizontal and vertical distributions obtained from reanalysis data (Supplementary Fig. S3). The integration was performed for 30 days, and the model outputs were averaged from days 26 to 30 after reaching their equilibrium state.

## Supplementary Information

Supplementary Information.

## Data Availability

The ERA5 reanalysis data used for description in the study are available at "Copernicus Climate Change Service Climate Data Store (CDS)" via https://cds.climate.copernicus.eu/cdsapp#!/search?type=dataset (Hersbach et al., 2020). AMO index is available at "The Climate Data Guide: Atlantic Multi-decadal Oscillation (AMO)" via https://climatedataguide.ucar.edu/climate-data/atlantic-multi-decadal-oscillation-amo (Enfield et al., 2001). The LBM code is available from http://ccsr.aori.u-tokyo.ac.jp/~lbm/sub/lbm.html.
