# Supplementary material for: East Asian heatwaves driven by Arctic-Siberian warming
Source: Sci Rep. 2022 Oct 27;12:18025. doi: 10.1038/s41598-022-22628-9 (PMC9614011; doi:10.1038/s41598-022-22628-9)
Supplement: Supplementary file 1 — Supplementary Information. [file 41598_2022_22628_MOESM1_ESM.docx]

**Supplementary Information**

# East Asian heatwaves driven by Arctic-Siberian warming

Jeong-Hun Kim^1,2^, Seong-Joong Kim^2^*, Joo-Hong Kim^2^,
Michiya Hayashi^3^, Maeng-Ki Kim^1*^

^1^Department of Atmospheric Sciences, Kongju National University, Gongju 32588, Republic of Korea

^2^ Division of Atmospheric Sciences, Korea Polar Research Institute, Incheon 21990, Republic of Korea

^3^ Earth System Division, National Institute for Environmental Studies, Tsukuba, Ibaraki, 305-8506, Japan

*Corresponding Author:
Dr. Seong-Joong Kim ([seongjkim@kopri.re.kr](mailto:seongjkim@kopri.re.kr)) and Prof. Maeng-Ki Kim ([mkkim@kongju.ac.kr](mailto:mkkim@kongju.ac.kr))

Supplementary Figure S1. Regression pattern associated to the SVD 1st mode. Regressed geopotential height at 250 hPa (GPH250; shading; units: m) and wave activity flux (WAF; vector; units: m^2^/s^2^) on time coefficient of SVD mode 1 (TC1). The hatched patterns indicate statistically significant at a 90% confidence level. The gray vectors indicate all WAF vectors, and the black vectors indicate statistically significant at a 95% confidence level.

Supplementary Figure S2. Diurnal variation in the ASP surface. Diurnal variation of the SAT (red lines; units: °C), 2m specific humidity (Q2m; blue lines; units: g/kg), and evaporation (green lines; units: mm) in the ASP. Solid and dashed lines indicate the composite of the HW-ASP and NHW-ASP, respectively. The red shading indicates a total variation of SAT in the HW-ASP.

Supplementary Figure S3. Steady forcing distribution of LBM experiments. The horizontal (bottom panel) and vertical (upper panel) distribution of the prescribed vorticity tendency (units: 1/s^2^; left panel) and diabatic heating (Q1; units: K/day; right panel) forcings were used in the LBM experiment. The vorticity tendency at 0.2945 sigma level and Q1 at 0.995 sigma level are shown in c and d. The vertical profiles in (a) and (b) are averaged in the ASP region (black boxes in c and d).

Supplementary Figure S4. Results of LBM experiments. The horizontal (upper panel) and cross-section (bottom panel) distribution of atmospheric responses to the vorticity forcing (left panel) and diabatic heating (right panel) forcings in the LBM experiment. These steady forcings in LBM are derived from the daily relative vorticity tendency and diabatic heating over the ASP region from the surface level to 10 hPa at the temporal averaging of −3 to onset days in the P1 and P2 periods in Fig. 4(a) and 4(b).

Supplementary Figure S5. Empirical Orthogonal Function (EOF) modes of GPH250 over the Northern hemisphere. (a–b) EOF pattern and (c–d) the corresponding PC time series of GPH250 over the Northern Hemisphere. The second mode indicates the CGT pattern. The solid green line in (d) shows a CGT index (CGTI). The correlation coefficient between the second mode of PC time series and CGTI is 0.32, which is statistically significant at the 95% confidence level.
